# RNAs Containing Modified Nucleotides Fail To Trigger RIG-I Conformational Changes for Innate Immune Signaling

**DOI:** 10.1128/mBio.00833-16

**Published:** 2016-09-20

**Authors:** Ann Fiegen Durbin, Chen Wang, Joseph Marcotrigiano, Lee Gehrke

**Affiliations:** a Program in Virology, Division of Medical Sciences, Harvard University, Boston, Massachusetts, USA; b Department of Chemistry and Chemical Biology, Center for Advanced Biotechnology and Medicine, Rutgers University, Piscataway, New Jersey, USA; c Institute for Medical Engineering and Science, Massachusetts Institute of Technology, Cambridge, Massachusetts, USA; d Department of Microbiology and Immunobiology, Harvard Medical School, Boston, Massachusetts, USA

## Abstract

Invading pathogen nucleic acids are recognized and bound by cytoplasmic (retinoic acid-inducible gene I [RIG-I]-like) and membrane-bound (Toll-like) pattern recognition receptors to activate innate immune signaling. Modified nucleotides, when present in RNA molecules, diminish the magnitude of these signaling responses. However, mechanisms explaining the blunted signaling have not been elucidated. In this study, we used several independent biological assays, including inhibition of virus replication, RIG-I:RNA binding assays, and limited trypsin digestion of RIG-I:RNA complexes, to begin to understand how RNAs containing modified nucleotides avoid or suppress innate immune signaling. The experiments were based on a model innate immune activating RNA molecule, the polyU/UC RNA domain of hepatitis C virus, which was transcribed *in vitro* with canonical nucleotides or with one of eight modified nucleotides. The approach revealed signature assay responses associated with individual modified nucleotides or classes of modified nucleotides. For example, while both *N-*6-methyladenosine (m6A) and pseudouridine nucleotides correlate with diminished signaling, RNA containing m6A modifications bound RIG-I poorly, while RNA containing pseudouridine bound RIG-I with high affinity but failed to trigger the canonical RIG-I conformational changes associated with robust signaling. These data advance understanding of RNA-mediated innate immune signaling, with additional relevance for applying nucleotide modifications to RNA therapeutics.

## IMPORTANCE

The innate immune system provides the first response to virus infections and must distinguish between host and pathogen nucleic acids to mount a protective immune response without activating autoimmune responses. While the presence of nucleotide modifications in RNA is known to correlate with diminished innate immune signaling, the underlying mechanisms have not been explored. The data reported here are important for defining mechanistic details to explain signaling suppression by RNAs containing modified nucleotides. The results suggest that RNAs containing modified nucleotides interrupt signaling at early steps of the RIG-I-like innate immune activation pathway and also that nucleotide modifications with similar chemical structures can be organized into classes that suppress or evade innate immune signaling steps. These data contribute to defining the molecular basis for innate immune signaling suppression by RNAs containing modified nucleotides. The results have important implications for designing therapeutic RNAs that evade innate immune detection.

## INTRODUCTION

The pattern recognition receptors of the innate immune system must distinguish the chemical patterns of “self” versus “non-self” molecules. Three homologous helicases, retinoic acid-inducible gene I (RIG-I), melanoma differentiation-associated protein 5 (MDA5), and laboratory of genetics and physiology 2 (LGP2), constitute the cytosolic RIG-I-like receptors (RLRs). Members RIG-I and MDA5 have similar domain architectures, including tandem N-terminal caspase activation and recruitment domains (CARDs) that participate in signaling, a DExD/H-box helicase domain with RNA binding and ATP hydrolysis activity, and a C-terminal domain (CTD). While MDA5 detects long double-stranded RNA, the salient chemical feature of RIG-I ligands is a 5′ triphosphate (5′ppp) (1, 2) or diphosphate (3). Other reported RIG-I ligands include the panhandle structures of viral genomic RNA (reviewed in reference 4) and uridine-rich sequences (5–7).

In the absence of ligand RNA, both RIG-I and MDA5 adopt an autorepressed conformation, with subsequent activation elucidated by structural studies (reviewed in reference 8). The 5′ppp of an RNA ligand is bound by the RIG-I CTD, enabling subsequent helicase:RNA interactions and ATP binding. This induces a RIG-I conformational change that releases the CARDs for K63-linked polyubiquitination (9) and binding of unanchored K63-linked ubiquitin chains (10). This activated RIG-I conformer then engages the adaptor, mitochondrial antiviral signaling protein (MAVS), to mediate activation of transcription factors and interferon-stimulated gene (ISG) induction. Genetic mutations of MDA5 or RIG-I can produce autoimmune pathologies (reviewed in reference 11). Both MDA5 and RIG-I are reported to use kinetic discrimination or “proofreading” of self-ligands versus non-self-ligands by ATP hydrolysis (12–16).

Several studies support the hypothesis that the type and density of RNA nucleotide modifications regulate the innate immune distinction between cellular and pathogen RNA. Recent reports suggest that the *N*-7-methylation of Cap-0 (m7GpppNpNpN) is insufficient to suppress RIG-I signaling activation and that the ribose 2′ O-methylation of Cap-1 (m^7^GpppNmpNpN) is critical (17, 18). Indeed, Cap-1 methylation also prevents mRNA detection both by MDA5 (19) and by the interferon-induced protein with tetratricopeptide repeats (IFIT) family of restriction factors (20). Post-transcriptional modifications of internal RNA nucleotides also impact innate immune detection. Karikó and colleagues first reported that signaling through membrane-bound RNA-sensing Toll-like receptors was significantly diminished in response to RNA ligands containing modified internal nucleotides (21). MDA5 detection of self-double-stranded RNA (self-dsRNA) is blocked by A-to-I modification catalyzed by adenosine deaminase acting on RNA (ADAR1) (reviewed in reference 22). Mutations in RIG-I and MDA5 are also associated with autoimmune syndromes (11), where the receptors may be activated in the absence of viral infection, for example, by endogenous RNAs. Previous reports also suggest that the RIG-I response to RNA ligands is damped by nucleotide modifications (6, 23). Despite the accumulating evidence that nucleotide modifications of cellular RNA can serve an immunoevasive role in preventing autoimmune activation, the mechanism(s) of pattern recognition receptor signaling suppression is undetermined.

Among the 100+ known nucleotide modifications (24), several modifications previously assumed to exist only in ribosomal and transfer RNAs have recently been mapped across the transcriptome of mammalian cell lines, yeast, and bacteria, with their enrichment patterns providing clues to their biological function in other RNA species. The density of base modifications observed in cellular RNA is reportedly low; for example, experimental evidence suggests an average of 3 to 5 sites of *N-*6-methyladenosine (m6A) modification per mRNA and modification at a substoichiometric frequency at each site (25). However, the biological impact of a modification may be determined more by location than by density, as suggested by the specific enrichment patterns observed in transcriptome-wide mapping of m6A, 5-methylcytidine (5mC), and pseudouridine (Ψ). Since it has been mapped across the mammalian, yeast, plant, and bacterial transcriptomes, the finding of m6A enrichment near the cap and stop codon of an mRNA supports functional evidence of m6A regulating RNA stability, splicing, and translation (reviewed in reference 26). Distinct from DNA methylation, 5mC in the human transcriptome was enriched in untranslated regions and Argonaut binding sites of mRNA and was also observed in noncoding RNAs (27). Pseudouridine (Ψ) has been mapped in the human and yeast transcriptome and is dynamically regulated by environmental signals (reviewed in reference 28). Human-designed 2′ fluoro-deoxyribonucleotides (2FdN), when introduced by *in vitro* transcription or in chemically synthesized small interfering RNAs (siRNAs), confer nuclease resistance and immunoevasive characteristics (29, 30).

Here, we use a well-established RIG-I-activating RNA ligand, the 106-nucleotide (nt) polyU/UC sequence derived from the 3′ untranslated region (UTR) of hepatitis C virus (5, 6), as a platform for exploring the immunosuppressive potential of several nucleotide modifications. We present evidence suggesting that m6A, Ψ, *N*-1-methylpseudouridine (mΨ), 5mC, 5-hydroxymethylcytidine (5hmC), 5-methoxycytidine (5moC), and 2′ fluoro-deoxyribose modifications (2′ fluoro-deoxyuridine [2FdU] and 2′ fluoro-deoxycytidine [2FdC]) individually suppress RIG-I responses to the polyU/UC RNA ligand. The results of several distinct experimental approaches suggest that RNAs containing modified nucleotides impact multiple steps early in the RIG-I signaling pathway. Strikingly, limited trypsin digestion experiments revealed that RNAs containing Ψ or *N*-1-methylpseudouridine (mΨ) bound to RIG-I but failed to trigger conformational changes associated with RIG-I activation. These data advance our understanding of RNA-mediated innate immune signaling, with further significance for applying nucleotide modifications to RNA therapeutics (31).

## RESULTS

### ***In vitro*** transcription of RNA containing modified nucleotides (**RNA*mod*).**

**FIG 1:**
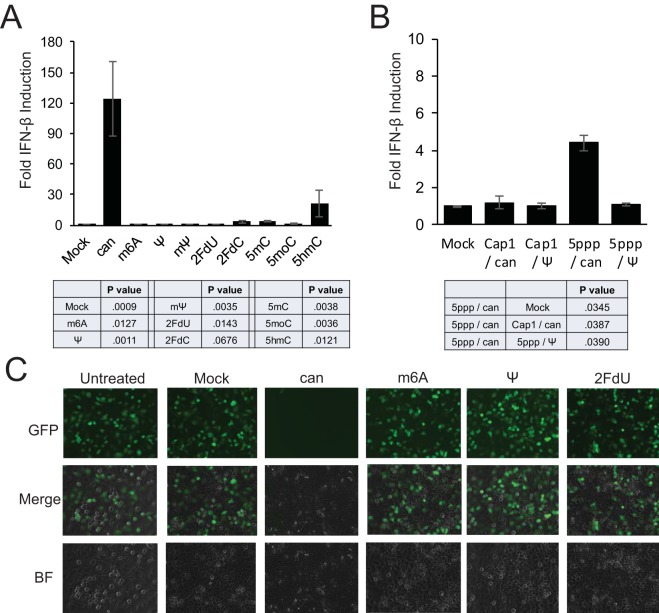
RNA*mod* and RIG-I-mediated IFN-β induction. (A) Huh7 cells were first transfected with luciferase reporter plasmids and then later mock transfected or transfected with 400 ng of polyU/UC RNA containing canonical nucleotides (can) or polyU/UC RNA containing the indicated modified nucleotides. (B) Following reporter plasmid transfection, Huh7 cells were transfected with EGFP mRNA containing a Cap-1 structure (Cap1) or 5′ppp terminus (5ppp) and canonical (can) nucleotides or pseudouridine substitution (Ψ). (A and B) After an additional 16 to 24 h of incubation, cell lysates were analyzed for IFN-β-promoter-driven firefly luciferase activity, and the data were normalized to constitutively produced *Renilla* luciferase values to control for transfection efficiency and then normalized to the mock-treated control condition. Error bars represent the standard deviations of results determined among technical triplicates, with results of a representative experiment shown. The statistical table reports the *P* values of results of paired two-tailed *t* tests comparing fold IFN responses between (A) canonical polyU/UC RNA and indicated modified RNAs or between (B) the indicated mRNAs, from multiple independent experiments. Additional data are provided in Fig. S1 and S2 in the supplemental material. (C) Huh7 cells were transfected with 400 ng polyU/UC RNA containing the indicated modified nucleotides. After 16 h of incubation, cells were infected with VSV-GFP at an MOI of 3. At 6 h postinfection, GFP expression was visualized by live-cell microscopy. Additional data are provided in Fig. S3 and S4 in the supplemental material. BF, bright field.

**FIG 2:**
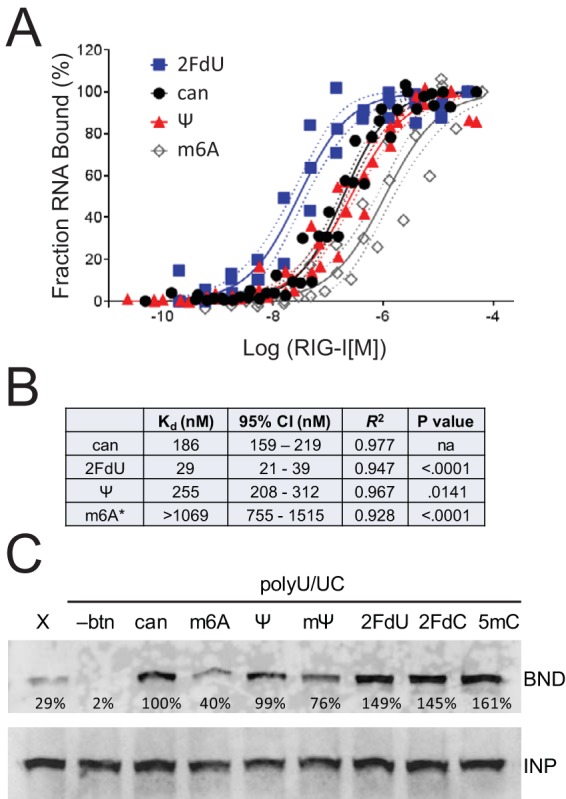
RNA*mod* and RIG-I binding affinity. (A) Radiolabeled polyU/UC RNA was incubated with purified recombinant RIG-I to allow complex formation and then applied to a nitrocellulose membrane filter, which retains RNA-protein complexes, while unbound RNA passes through the membrane. The fraction of bound RNA was normalized to the maximum observed signal. Combined data from at least three independent experiments per ligand are presented with solid lines indicating the best-fit nonlinear regression and dashed lines indicating 95% confidence intervals. (B) Calculated equilibrium binding dissociation constants (*K_d_*) derived from data shown in panel A, including 95% confidence interval, goodness of fit (*R^2^*), and statistical test (*P* value), demonstrating a significant difference between the two curve parameters *K_d_* and slope for each comparison of RNA*mod* versus RNA*can*. For the m6A data (indicated by an asterisk [*]), the experimental maximum observed signal was normalized to 100% RNA bound, although a binding plateau was not observed. Therefore, the accurate RIG-I:RNA*m6A* binding constant is likely higher (lower affinity) than that presented. Additional data are provided in Fig. S5 in the supplemental material. (C) Biotinylated polyU/UC RNA with the indicated modified nucleotides was added to Huh7 cell lysate. Negative controls included biotinylated X-RNA (X) and nonbiotinylated polyU/UC (-btn). RIG-I:RNA complexes that were captured with streptavidin-conjugated paramagnetic beads (BND) were detected by Western blotting with anti-RIG-I, relative to the RIG-I in 10% input (INP). The signal was quantified in the BND fraction relative to the INP fraction and normalized to canonical RNA (100%).

**FIG 3:**
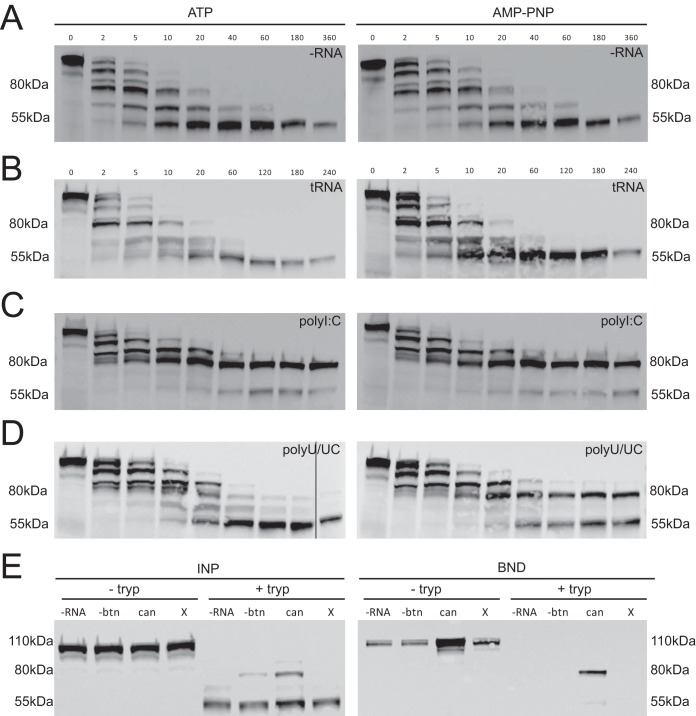
RIG-I:RNA conformation as probed by limited trypsin digestion time course. (A to D) RIG-I fragments were detected by Western blotting with a monoclonal antibody to the helicase domain. Cell extracts were incubated with trypsin in the absence (A [–RNA]) or presence of RNA ligands, including nonbinding control yeast tRNA (B [tRNA]), polyI:C (C), or polyU/UC (D), and with 1 mM ATP or AMP-PNP. Aliquots were removed from the reaction at various time points (minutes) post-addition of trypsin. Additional data are provided in Fig. S6 in the supplemental material. (E) Biotinylated RNAs were used in pulldown experiments to test for RNA binding by the 80-kDa and 55-kDa RIG-I fragments. Trypsin digests (+ tryp) and control lysates (− tryp) were incubated without RNA (-RNA), with nonbiotinylated polyU/UC RNA (-btn), with biotinylated polyU/UC RNA (can), or with biotinylated X RNA (X) in the presence of AMP-PNP. After 1.5 h, trypsin digestions were quenched by adding protease inhibitor and incubated with streptavidin paramagnetic beads. RIG-I present in the bead-bound fraction (BND) versus the input fraction (INP) was detected by Western blotting.

**FIG 4:**
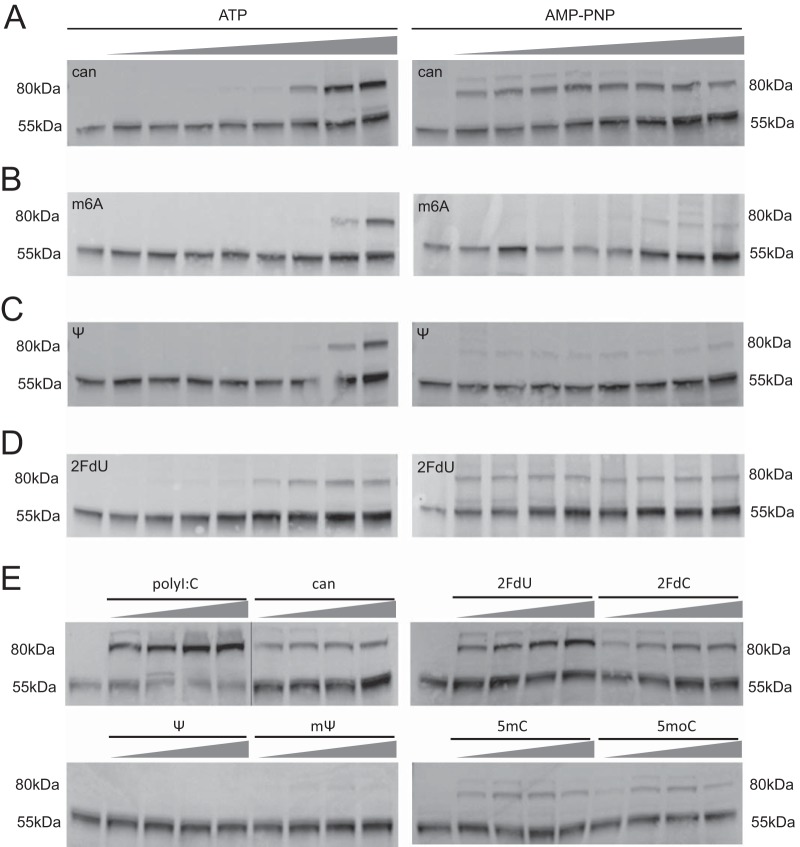
RNA*mod* and RIG-I trypsin sensitivity. (A to D) Digestion of 293T cell lysate for 2 h in the presence of polyU/UC RNA with the indicated modifications or canonical nucleotides (can), at increasing polyU/UC RNA concentrations (0, 12.5, 25, 50, 100, 200, 400, 800, and 1,600 nM), in the presence of 2 mM ATP or AMP-PNP. Data represent results of Western blotting for 55-kDa and 80-kDa fragments of RIG-I with anti-helicase antibody. (E) Digestions of 293T cell lysate performed for 1.5 h in the presence of AMP-PNP and polyU/UC RNA with the indicated modifications, at increasing concentrations (0, 33, 100, 300, and 900 nM), or mass equivalent of polyI:C.

Before performing assays to probe mechanisms of innate immune signaling suppression by modified nucleotides (see Fig. 2 to 4), we first performed validation experiments to define RNA quality and functional activity in cell-based assays (Fig. 1; see also Fig. S1 to S4), which included expanding our prior work (6) to include five additional modified nucleotides. Modified nucleotide triphosphates were substituted for canonical nucleotide triphosphates in T7-polymerase transcription reactions (see Fig. S1B). Transcription products were routinely tested for size and homogeneity by capillary gel electrophoresis (see Fig. S1C). To control for dsRNA (32) in our preparations, we adapted a dot blot method from Karikó and colleagues (33), using a monoclonal antibody clone J2 that recognizes dsRNA longer than 40 bp (34). The results demonstrated that the polyU/UC RNAs used in our assays were free of detectable long dsRNA (see Fig. S1D). RNA batches that failed to meet this criterion were purified by high-performance liquid chromatography (HPLC), using a protocol adapted from Karikó and colleagues (33), and were then retested for dsRNA content.

### RNA*mod* evasion of RIG-I-mediated IFN-β induction.

Purified polyU/UC RNAs containing canonical nucleotides (RNA*can*) or modified nucleotides (RNA*mod*) were then assayed in a panel of cell-based assays (Fig. 1) to test for differences in innate immune stimulation. A interferon beta (IFN-β) promoter-driven luciferase reporter assay was used previously to characterize RNA*can* and RNA*mod* signaling using polyU/UC RNA (6). Related approaches were used here to validate current experimental conditions and to provide direct functional comparisons with five additional modified nucleotides transcribed into polyU/UC (Fig. 1A) or mRNA (Fig. 1B). Distinct from our previous work (6) where RIG-I was expressed from a transfected plasmid, the innate immune signaling approaches described here reflect endogenous cellular RIG-I activity instead of overexpressed RIG-I. Huh7 cells were cotransfected with the IFN-β reporter plasmid and a constitutive luciferase expression transfection control plasmid. Cells transfected with RNA*can* or RNA*mod* were analyzed at 16 to 24 h posttransfection (hpt). As shown in Fig. 1A, the polyU/UC RNA containing canonical nucleotides (can) activated robust IFN-β promoter induction, in agreement with previous published reports (5, 6, 35). RNA*mod* containing other modified nucleotides (m6A, Ψ, mΨ, 2FdU, 2FdC, 5mC, 5moC, and 5hmC) stimulated significantly less IFN-β reporter activity than RNA*can* (Fig. 1A). To determine if signal suppression would be observed using a longer RNA with a lower percentage (10.3%) of uridine content, the assay was repeated with mRNA (~1,000 nt) encoding enhanced green fluorescent protein (EGFP) (Fig. 1B). The highest interferon activation was observed using the uncapped mRNA transcript that was transcribed using canonical nucleotides (5ppp/can), consistent with 5ppp being an important RIG-I stimulatory signal (1, 2). However, complete substitution of pseudouridine for uridine (5ppp/Ψ) also reduced the IFN-β response to the 5ppp-containing mRNA (Fig. 1B). As predicted, the 5ppp activation signal was also diminished significantly in the interferon induction assay using RNA containing a Cap-1 structure. EGFP-expressing cells were observed by live-cell fluorescence when the Cap-1/can-EGFP and Cap-1/Ψ-EGFP mRNAs were transfected, while cells receiving the 5′ppp-containing mRNAs (5ppp/can and 5ppp/Ψ) did not show detectable fluorescence (data not shown), reflecting the known importance of the 5′ cap structure for mRNA translation. The absence of innate immune signaling observed using RNAs containing modified nucleotides could be explained by a complete failure of the RNA*mod* to enter the cells. However, the literature suggests that RNAs containing modified nucleotides retain function upon transfection with commercial cationic lipid reagents (see, for example, references 36 and 37); moreover, the observed EGFP expression from mRNA containing 10.3% pseudouridine demonstrated successful RNA transfection. The results presented here strongly suggest that RNAs containing modified nucleotides suppress or evade innate immune stimulation.

To further validate our experimental system, two control experiments were performed to demonstrate that the IFN-β signaling assays reflected specific RNA activation of the RIG-I innate immune receptor. RIG-I recognizes the RNA 5′ triphosphate (1, 2), and converting 5′ppp RNA to 5′OH terminus RNA with calf intestinal phosphatase (CIP) prevents RIG-I CTD:RNA interaction and subsequent signaling activation (1). We observed that subjecting the polyU/UC RNAs to CIP treatment reduced Huh7 cell IFN-β reporter responses (see Fig. S2A in the supplemental material), consistent with a RIG-I-mediated response. Huh7.5 cells were used as a second control to demonstrate RIG-I-specific receptor activation. Huh7.5 cells express RIG-I protein with a T55I point mutation in the RIG-I CARDs, which blocks downstream signaling (38), including blocking the response to ligands such as polyU/UC (39). Indeed, the Huh7.5 cells lacked IFN-β induction responses to canonical and modified polyU/UC RNA but retained responses to long dsRNA, most likely detected by the MDA5 receptor (see Fig. S2A). In addition, endogenous ISG expression in response to the transfected polyU/UC RNA was observed using Huh7 cells but not Huh7.5 cells (see Fig. S2B and C). Taken together, the data presented in Fig. 1 and in Fig. S1 to S3 strongly suggest (i) that RNA transcript quality was carefully controlled, (ii) that the polyU/UC ligand indeed signals through the cytoplasmic RIG-I pattern recognition receptor to activate an IFN-β reporter, as well as endogenous ISGs expression, and (iii) that 106-nt polyU/UC RNA and 996-nt EGFP mRNA transcribed with modified nucleotides significantly suppress RIG-I signal transduction compared with RNAs containing canonical nucleotides.

### RNA*mod* evasion of RIG-I antiviral signaling.

To verify that the observed IFN-β induction and ISG expression indeed reflected an antiviral state, Huh7 cells were challenged with vesicular stomatitis virus (VSV). Briefly, Huh7 cells were first transfected with RNA to activate RIG-I-dependent signaling. RNA*can* transfection was expected to activate interferon expression and therefore to reduce VSV replication. At 16 h posttransfection, cells were washed and infected with recombinant VSV encoding a GFP reporter (VSV-GFP)*.* Huh7 cells challenged with VSV-GFP were analyzed at 6 h postinfection (hpi) with fluorescence microscopy imaging (Fig. 1C). Infection was quantified by flow cytometry of unfixed cells to detect native GFP fluorescence (see Fig. S3A in the supplemental material). Cells receiving no polyU/UC RNA during pretreatment were 70% to 95% GFP positive (GFP^+^), reflecting a permissive state for VSV replication. Cells pretreated with RNA*can* were only 1% to 5% GFP^+^, indicating that viral replication was suppressed. Conversely, pretreatment with RNA*mod* did not protect cells from VSV-GFP infection. Similar results were observed in a dengue virus (DenV) challenge, as assayed by flow cytometry (see Fig. S3B). These results are consistent with the luciferase reporter data (Fig. 1A) and ISG induction data (see Fig. S2C), demonstrating that RNA*can* signals through RIG-I to induce interferon expression and an antiviral state, while RNA*mod* is unable to stimulate RIG-I-mediated antiviral signaling.

One additional control was performed before proceeding to mechanistic studies. Differential innate immune stimulation by modified RNA (RNA*mod*) could potentially be explained by differential RNA stability. We therefore analyzed RNA stability in cell extracts. By adding radiolabeled polyU/UC RNA to 293T cell extracts, we observed that RNA containing Ψ (RNAΨ) and RNA containing m6A (RNA*m6A*) had a half-life similar to that of RNA with canonical nucleotides (RNA*can*), while RNA*2FdU* was hyperstable (see Fig. S4 in the supplemental material). Indeed, the 2FdU ribose modification has been previously reported to confer nuclease resistance (29). These data suggest that reduced RIG-I signaling responses to modified RNA are not explained simply by differential RNA*mod* stabilities. Taken together, the data presented in Fig. 1 and in Fig. S1 to S4 define a robust experimental system for use in further mechanistic studies (Fig. 2 to 4) of RNA*can* and RNA*mod*.

### RNA:RIG-I binding affinity.

The first mechanistic analysis tested the hypothesis that RIG-I signaling correlates directly with RNA:RIG-I binding affinity. We used a filter binding approach to quantify ^32^P-radiolabeled RNA*can* and RNA*mod* binding to increasing concentrations of purified recombinant RIG-I protein. Binding specificity was demonstrated by competitive binding experiments, where radiolabeled RNA*can* was incubated with increasing concentrations of nonradiolabeled competitor RNAs (see Fig. S5 in the supplemental material).

The equilibrium dissociation constants revealed by the filter binding experiments strongly suggest that nucleotide modifications have differential effects on RIG-I:RNA affinities (Fig. 2A and B). RNA*2FdU* bound RIG-I with the highest affinity, with an approximate dissociation constant of 29 nM (Fig. 2B). RNA*can* and RNA*Ψ* had approximate dissociation constants of 186 nM and 255 nM, respectively, which were statistically significantly distinct (Fig. 2B). Alternatively, two independently transcribed RNA*m6A* preparations each bound RIG-I with much lower affinity; moreover, the binding did not plateau, even at the highest RIG-I concentrations that were feasible in the experimental design. Therefore, the maximum observed RNA*m6A* binding value was calibrated to 100% in order to generate a nonlinear regression, revealing a dissociation constant greater than 1,069 nM (Fig. 2B).

The experiments represented in Fig. 2A were performed using bacterially expressed recombinant RIG-I protein. We complemented this approach with a pulldown method that instead used endogenously expressed RIG-I in Huh7 cell extract. PolyU/UC RNA was transcribed with biotin-11–CTP and incubated with Huh7 cell extract. RIG-I that coisolated with the biotinylated RNA via paramagnetic streptavidin beads was detected by Western blotting. Negative-control RNA*can* transcribed without biotin did not isolate RIG-I, confirming that the RIG-I signal in the bead-bound fraction requires biotin-based RNA pulldown. As a second control, we used biotinylated 5′ppp X-region RNA (98 nt), also derived from the 3′UTR of hepatitis C virus (HCV) and previously observed to have lower RIG-I affinity than 5′ppp polyU/UC RNA (5, 6). We observed a weak but detectable RIG-I interaction with biotinylated X-RNA, as expected (Fig. 2C). PolyU/UC RNA*m6A* pulled down less RIG-I than RNA*can*, in agreement with the quantitative filter binding assay. While the pseudouridine modifications (Ψ and mΨ) had limited impact on RIG-I pulldown, the 2′ fluoro-deoxyribose and 5mC modifications appeared to enhance RIG-I binding affinity compared to the RNA*can* results, also in agreement with the quantitative filter binding assay.

Taken together, the results from the two independent RIG-I:RNA binding assays (Fig. 2) suggest that signal transduction intensity does not necessarily correlate directly with RIG-I:RNA binding affinity (Fig. 1 and 2). These observations were unexpected because each of the RNAs containing the nucleotide modifications failed to activate RIG-I signaling in the IFN-β reporter assay and the antiviral signaling assay (Fig. 1). These data suggest that mechanisms of innate immune suppression are not uniform among nucleotide modifications, motivating additional experiments to define the relevant mechanisms.

### Limited trypsin digestion of RIG-I:RNA complexes.

As described previously, productive RNA:RIG-I binding and signaling are accompanied by RIG-I conformational changes that release the CARDs for K63-linked polyubiquitination (9) and binding of unanchored K63-linked ubiquitin chains (10). We hypothesized that RNAs containing certain modifications, such as pseudouridine, may bind to RIG-I without triggering the protein conformational changes necessary for downstream signaling. To test this hypothesis, we adapted a limited trypsin digestion protocol that had been used previously to assay the conformational states of RNA bound to recombinant RIG-I protein (35, 40–43). While previous reports used limited tryptic digestions to assay the conformation of bacterially expressed recombinant RIG-I (5, 35, 41, 43, 44), we adapted the digestion method to use cell extracts with mammal-expressed RIG-I. We performed protease experiments by adding polyU/UC RNAs to cell lysates prepared from 293T cells containing a chromosomally inserted construct for doxycycline-inducible human RIG-I overexpression.

Limited trypsin digestions were performed in the presence of ATP or of a nonhydrolyzable ATP analogue, adenylyl-imidodiphosphate (AMP-PNP). AMP-PNP promotes RIG-I domain compaction (8) and stabilizes the ternary complex of RIG-I:RNA:AMP-PNP without the ATP hydrolysis that is reported to dissociate RNA from the helicase domain (13, 14). Briefly, RNA and ATP or RNA and AMP-PMP were added to cell lysates and the mixtures were incubated at room temperature to allow RIG-I:RNA complexes to form. Next, trypsin was added to the reaction mixture to partially digest the complexes, and the protease activity was halted by adding SDS gel sample buffer and boiling. Tryptic protein fragments were separated by SDS-PAGE, and RIG-I-specific fragments were detected by immunoblotting with a commercial anti-helicase domain antibody raised against RIG-I amino acids (aa) 201 to 713.

Control experiments were performed to demonstrate signal specificity and to define protease digestion parameters (Fig. 3A to D), prior to testing the RNAs containing modified nucleotides (Fig. 4). Overall, the results of the RIG-I trypsin digestions focused our attention on 80-kDa and 55-kDa RIG-I tryptic fragments that were differentially trypsin resistant depending on the RNA ligand used. In the absence of exogenous RNA ligand (-RNA) and in the presence of negative-control yeast tRNA, a relatively stable 55-kDa RIG-I fragment was observed (Fig. 3A and B). We interpret the 55-kDa RIG-I fragment as representing the trypsin sensitivity of autorepressed RNA-free RIG-I conformation. This 55-kDa pattern was observed in the presence of ATP or in the presence of AMP-PNP, consistent with the assumption that ATP hydrolysis does not regulate the autorepressed conformation of RIG-I (45).

An 80-kDa RIG-I fragment remained trypsin resistant when either dsRNA mimic poly(I ⋅ C) (polyI:C) or polyU/UC RNA was added to the lysates (Fig. 3C and D). We interpret the 80-kDa fragment to represent the trypsin resistance of the RNA-bound RIG-I in the activated conformation (8). Digestions performed with the same RNAs in Huh7 cell lysate also generated 80-kDa and 55-kDa RIG-I fragments (see Fig. S6 in the supplemental material), suggesting that the patterns were not an artifact of the RIG-I construct cloned into the doxycycline-inducible 293T cell line. Interestingly, visualizing the 80-kDa RIG-I tryptic fragment under the polyU/UC RNA conditions required AMP-PNP, suggesting that ATPase activity dissociates the RNA ligand and returns RIG-I to the autorepressed conformation (Fig. 3D). We therefore hypothesized that the 80-kDa fragment represented the trypsin resistance of a RIG-I:RNA complex involving helicase domain interactions.

To test for RNA binding activity of the RIG-I tryptic fragments, we performed trypsin digests in the presence of biotinylated polyU/UC RNA. While both 55-kDa and 80-kDa RIG-I fragments were formed during the digestion (Fig. 3E [INP]), only the 80-kDa fragment was captured with biotinylated polyU/UC RNA during the streptavidin bead pulldown (Fig. 3E [BND]). This observation suggests that the 80-kDa RIG-I fragment indeed represents the trypsin resistance of a RIG-I:RNA complex.

### RNA*mod* impact on RIG-I:RNA trypsin sensitivity.

**FIG 5:**
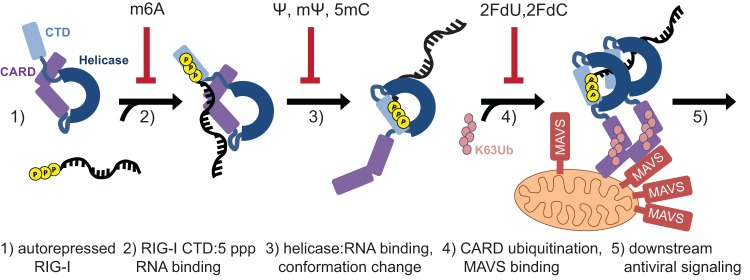
Model of RIG-I interaction with RNA*can* and RNA*mod*. (Step 1) RIG-I is in an autorepressed conformation in the absence of ligand. (Step 2) 5′ppp RNA binds the RIG-I CTD. RNA*m6A* binds RIG-I with lower affinity than RNA*can*. (Step 3) The RIG-I helicase domain binds the RNA, triggering a protein conformational change. RNAs containing Ψ, mΨ, or 5mC fail to induce RIG-I conformational change. (Step 4) The released CARDs of the activated RIG-I:RNA complex are ubiquitinated and signal downstream. This process is hypothesized to be inefficient for RIG-I:RNA*2FdN*, possibly due to ATPase negative regulation of RNA binding. Ubiquitinated RIG-I induces MAVS aggregation on the surface of the mitochondria. (Step 5) Mitochondrial complexes mediate downstream antiviral signaling. Among the RNAs tested, we observed that only RNA*can* triggered RIG-I antiviral signaling.

We next applied the limited trypsin digest assay to define RIG-I conformational states under conditions of binding to RNA*mod*. The experimental conditions presented in Fig. 3A to D included a constant RNA concentration and a digestion time course. To extend this analysis, we tested trypsin sensitivity with RNA*mod* using a single reaction time point with increasing RNA concentrations (Fig. 4). Interestingly, the digestion patterns were similar across RNA*mod* ligands in assays performed in the presence of ATP; however, in the presence of AMP-PNP, we observed differential results with the 80-kDa RIG-I fragment (Fig. 4). This suggests that the RNA*mod* ligands differ in their propensity to bind the helicase domain and that they cooperatively bind ATP or the AMP-PNP nucleotide. We further expanded our analysis to test RNAs containing other nucleotide modifications, including *N*-1-methylpseudouridine (mΨ), 2′ fluoro-deoxycytidine (2′FdC), 5-methylcytidine (5mC), and 5-methoxycytidine (5moC). The data (Fig. 4E) demonstrate that nucleotide modifications with similar chemical structures yielded common results in stabilizing the 80-kDa RIG-I conformer. Both RNA*Ψ* and RNA*mΨ* failed to generate the 80-kDa fragment, while RNA*5mC* and RNA*5moC* supported weak 80-kDa fragment formation. Interestingly, the 2′ fluoro-deoxyribose RNAs triggered robust 80-kDa fragment formation with either uridine or cytidine bases (Fig. 4E). These patterns suggest that RIG-I interaction with particular modified nucleotides in the polyU/UC ligand enables or prevents the activating protein conformational change and thus defines a mechanism of suppression of antiviral signaling (Fig. 5).

## DISCUSSION

During a pathogen infection, innate immune activation is essential for mounting a protective response; however, the responses must be closely regulated to prevent autoimmune effects. Several groups have proposed that nucleotide modifications distinguish self-RNAs from non-self-RNAs, based on observations that nucleotide modifications suppress Toll-like receptor 3 (TLR3), TLR7, and TLR8 (21, 29), RIG-I (6, 23), and other components of the innate immune system (46, 57). The goal of our study was to begin to define the mechanism(s) by which chemically disparate base and ribose modifications suppress or evade RIG-I signaling.

Using a highly stimulatory RNA ligand derived from HCV genomic RNA (5, 6), rigorous *in vitro* transcription RNA quality control (see Fig. S1 in the supplemental material), and multiple assays of RIG-I signaling (Fig. 1 to 4), we have tested and compared unmodified RNA and RNAs containing one of eight nucleotide modifications. Overall, we found that polyU/UC RNAs transcribed with each nucleotide modification dramatically suppressed or failed to activate RIG-I antiviral signaling compared to polyU/UC RNA transcribed with canonical nucleoside triphosphates (NTPs) (Fig. 1). Further work using multiple assays (Fig. 2 to 4) unexpectedly revealed that the modified nucleosides have different effects on RIG-I binding and on the conformation of the RIG-I:RNA complex and thus have different mechanisms of signaling suppression.

The limited trypsin digest experiments provided key data that distinguished the modified nucleotide classes. We adapted previously reported methods (35, 41, 44, 47) to probe the conformations of RIG-I:RNA complexes, electing to use mammalian cell extracts instead of purified recombinant RIG-I. Both the 55-kDa RIG-I fragment and the RNA-induced 80-kDa RIG-I fragment in our extract assay were recognized by a monoclonal anti-helicase domain antibody (Fig. 4; see also Fig. S6) but not by an anti-CARD antibody (data not shown). The 80-kDa fragment was also captured with biotinylated-polyU/UC RNA in a streptavidin bead pulldown, demonstrating RNA binding function (Fig. 4E). These observations are consistent with the interpretation that the 80-kDa fragment represents RIG-I lacking the CARDs. Meanwhile, the 55-kDa fragment lacked RNA binding activity in the biotinylated RNA pulldown assay. Therefore, we hypothesize that the 55-kDa fragment corresponds to the RIG-I helicase domain lacking both the CTD and CARDs. A direct comparison of the partial tryptic digestion patterns reported here and by others (35, 41, 44, 47) is challenging because of the use of different protein sources (extracts versus purified recombinant protein) and different detection methods (Coomassie staining versus immunoblotting) and differences in the RNA ligands used. Nonetheless, the tryptic digestion results, which were determined using internally consistent experimental conditions and controls, provide compelling data on RIG-I conformational changes induced by RNAs containing modified nucleotides.

Analyzed by multiple assays (Fig. 1 to 4), RNAs were observed to affect early signal transduction events in a nucleotide modification-specific manner (Fig. 5). RNA*m6A*, which bound RIG-I with low affinity (Fig. 2), did not trigger the conversion to the activated RIG-I conformer in the trypsin digest assay (Fig. 4B). Adenosines comprise only 6 of the 106 polyU/UC RNA nucleotides (see Fig. S1A in the supplemental material); therefore, the per-nucleotide functional effects on RIG-I:RNA*m6A* binding affinity were dramatic. PolyU/UC RNA containing pyrimidine modifications (Ψ, mΨ, 5mC) bound RIG-I with affinity comparable to or greater than canonical RNA (Fig. 2) while failing to induce the AMP-PMP-stabilized conformational change(s) that was detected as the 80-kDa fragment in the trypsin digest assay (Fig. 4E).

RNAs containing the ribose modification (2FdU or 2FdC) yielded unexpected and yet mechanistically informative experimental results. RNA*2FdU* and RNA*2FdC* bound RIG-I with high affinity (Fig. 2) and efficiently triggered a partial trypsin digestion pattern that represented activated RIG-I (Fig. 4). Unexpectedly, the RIG-I binding and RIG-I conformational changes observed with RNA*2FdN* were not accompanied by antiviral signaling; rather, IFN reporter induction was not observed (Fig. 1). One explanation for the differential assay results is that RIG-I helicase domain ATPase activity was able to drive helicase dissociation from RNA*2FdN* to abort antiviral signaling in the cytosol, while the AMP-PNP present *in vitro* in the limited trypsin digest assay stabilized the activated RIG-I:RNA*2FdN* complex (Fig. 4D). Others have proposed RIG-I ATPase negative regulation of RNA binding as well (13–16).

The methods and observations presented here are relevant to designing therapeutic mRNAs and siRNAs containing modified nucleotides that enhance RNA stability and reduce immunogenicity (48). The present study was limited to reporting on two RNA ligands (106-nt polyU/UC and 996-nt EGFP mRNA), a single pattern recognition receptor (RIG-I), and a panel of eight nucleoside modifications amenable to *in vitro* transcription. Future work should probe the mechanisms by which modified RNAs of diverse sequences, lengths, and structures suppress signaling by RIG-I and other innate immune receptors and effectors (MDA5, TLRs, and IFIT-family proteins). Determination of an appropriate RNA ligand for chemical synthesis would enable exploration of additional RNA modifications, including locked nucleic acids, and a phosphorothioate backbone (49, 50).

In addition to the diversity of synthetic nucleotides, over 100 naturally occurring nucleotide modifications, some of which may have evolved roles in self-detection versus non-self-detection by the innate immune system, have been previously described (24). It is likely that pattern recognition receptors detect more than the mere presence or absence of certain modifications; instead, a nuanced combination of the double- or single-strandedness of the RNA, the location of the modification (proximity to the 5′ or 3′ RNA terminus), and the sequence context of the modification is likely important.

We also speculate that pathogen-derived RNA ligands may contain modifications that influence detection by RIG-I; for example, dengue virus encodes a methyltransferase that acts primarily on the viral RNA cap and yet also catalyzes 2′ O methylation of adenosine residues in viral RNA, tRNA, and rRNAs *in vitro* (51). While the ribose methylation of the viral cap is critical for evasion of innate immune detection, the biological role of internal 2′ O-methyladenosine is unclear. Interestingly, influenza virus (negative-sense RNA genome) and Rous sarcoma virus (RNA retrovirus) produce viral transcripts with m6A modifications (52, 53), presumably catalyzed by cellular nuclear methyltransferases. The function of modifications—such as m6A—in the stability, translation, and immunogenicity of viral RNAs has not been explored in detail. Continued research at the interface of innate immunology and RNA modification biology will provide insights into self-detection versus non-self-detection, applicable in designing nucleic acid drugs to improve human health.

## MATERIALS AND METHODS

### Cell culture and virus stocks.

Huh7 cells were from the P. Yang laboratory (Harvard Medical School). Huh7.5 cells were from the C. Rice laboratory (Rockefeller University). 293TdoxRIG-I cells were generated from Flp-In T-Rex HEK293T cells (Thermo Fisher) by E. Karayel (Austrian Academy of Sciences). These cells are induced with doxycycline (Sigma-Aldrich) (10 µg/ml; 16 to 24 h) for expression of C-terminal Strep-hemagglutinin (HA)-tagged RIG-I. Dengue virus serotype 2, strain 16681, was cultivated on C6/36 cells, and titers were determined on BHK cells by flow cytometry (54). Vesicular stomatitis virus expressing green fluorescent protein (VSV-GFP) was from the S. Whelan laboratory (Harvard Medical School).

### Plasmids and protein.

The IFN-β-promoter-driven firefly luciferase plasmid pIFN-β-luc was provided by J. Jung (University of Southern California). The constitutive thymidine-kinase-promoter-driven *Renilla* luciferase plasmid pTK-Renluc was provided by M. Gack (University of Chicago). Human recombinant N-terminal 6× His-SUMO RIG-I was expressed and purified as described previously (44).

### RNA transcription.

The poly U/UC sequence (6) was transcribed *in vitro* from a short dsDNA template of annealed oligonucleotides from IDT. Long (2.7-kb) dsRNA was derived from the VP2 gene of rotavirus, as previously described (55). The sequence of interest was PCR amplified with primers containing a SmaI linearization site and T7 promoter for transcription, as well as KpnI and BamHI sites for cloning into the pUC19 plasmid. PolyI:C (GE Healthcare) was reconstituted in water.

Each RNA was transcribed *in vitro* using T7 Scribe (CellScript Inc.) or Durascribe (Epicentre) kits, according to the manufacturer’s instructions. Modified RNA was transcribed by 100% replacement with one of the following modified NTPs: N6-methyladenosine (m6A), pseudouridine (Ψ), N1-methylpseudouridine (mΨ), 5-methylcytidine (5mC), 5-methoxycytidine (5moC), 5-hydroxymethylcytidine (5hmC) (Trilink), 2′ fluoro-deoxyuridine (2FdU), 2′ fluoro-deoxycytidine (2FdC) (from the Durascribe kit [Epicentre]). Radiolabeled RNA was transcribed in the presence of a 0.33 µM final concentration of [α-^32^P]CTP (EasyTides; PerkinElmer) and 2.5 mM nonradioactive CTP. The RNA concentration was determined by the use of a NanoDrop 200 spectrophotometer (Thermo), and RNA purity was analyzed by capillary electrophoresis on a TapeStation 2200 instrument (Agilent).

Messenger RNAs (996 nt) encoding enhanced green fluorescent protein (EGFP) were provided by TriLink BioTechnologies, where they were transcribed *in vitro* with canonical nucleotides or complete substitution of pseudouridine for uridine, followed by purification. Transcripts were chemically modified with a CleanCap cotranscriptional capping system. A liquid chromatography-mass spectrometry (LC-MS) capping assay resulted in an estimation of 94% to 97% efficiency of generating m^7^GpppN^m^pNpN (Cap-1) (data not shown). Capped mRNAs were phosphatase treated to remove any residual 5′ triphosphate termini. Capped and uncapped (5′ triphosphate-containing) control mRNAs were HPLC purified (33).

### Dual-luciferase assay.

Huh7 cells were transfected in a 10-cm-diameter dish at 90% confluence with reporter plasmids (5 µg pIFN-β-luc and 500 ng pTK-RenLuc) using LipoJet (SignaGen Laboratories) or Lipofectamine 2000 (Life Technologies) according to the manufacturer’s protocol. The next morning, cells were released from the plate by mild trypsin digestion and reseeded at 1 × 10^5^ cells per well in a 24-well plate. Triplicate wells were transfected with RNA (50 to 1,000 ng/well) that was denatured at 85°C prior to complexing with LipoJet (SignaGen) or Lipofectamine (Invitrogen) 2000. At 16 to 24 h posttransfection, cell lysate in a 96-well plate was analyzed in a TriStar LB491 (Berthold) plate reader with automated injection of dual-luciferase reporter assay substrates (Promega). The fold induction of Firefly relative to *Renilla* was calculated per well, averaged across triplicates, and normalized to mock-treated wells.

### RIG-I induction of antiviral state.

Huh7 cells were seeded in a 24-well plate at 1 × 10^5^ cells/well or in a 12-well plate at 2 × 10^5^ cells/well. Cells were transfected with 200 to 600 ng/well of the indicated RNA ligand using Lipofectamine 2000 (Invitrogen) or LipoJet (SignaGen) or were mock transfected (with transfection reagent with no RNA) or left untreated. At approximately 16 hpi, cells were infected with VSV-GFP or mock infected in duplicate at a multiplicity of infection (MOI) of 3 or with dengue virus (DenV) at a MOI of 1. At 6 hpi, VSV-GFP-infected cells were imaged in an EVOS-fl microscope (Thermo Fisher). Then, VSV-GFP-infected cells were scraped in cold phosphate-buffered saline (PBS) for live-cell flow cytometry quantification of GFP^+^ cells. DenV-infected cells were fixed at 36 to 48 hpi, permeabilized, and stained with anti-DenV NS1 antibody (Abcam clone DN2) and a fluorescein-conjugated secondary antibody (Sigma). Flow cytometry was performed with a Guava flow cytometer with EasyCyte software (EMD Millipore).

### RIG-I:RNA filter binding assay.

Filter binding reactions were performed with ^32^P-radiolabeled RNA and recombinant purified RIG-I passed through a nitrocellulose filter, with RNA detected by scintillation counting. Data normalizations were performed as described previously (56). See Text S1 in the supplemental material for details.

### Biotinylated RNA pulldown.

Confluent 10-cm-diameter dishes of Huh7 cells were scraped in binding buffer (25 mM Tris-HCl [pH 7.5], 150 mM NaCl, 1.5 mM MgCl_2_) supplemented with 0.5% Triton X-100 and EDTA-free protease inhibitor (Roche). Binding reaction mixtures contained 2 µg of biotinylated RNA or nonbiotinylated RNA negative control and 500 to 750 µg of clarified lysate in a final volume of 120 µl. After 30 min, reaction mixtures were supplemented with 150 µl of streptavidin-conjugated paramagnetic bead slurry (Promega) and incubated at room temperature for 1 h with rocking. Beads and associated RNA:protein complexes were captured with a magnetic rack and washed in binding buffer. Beads were boiled in SDS-PAGE sample buffer, and Western immunoblot analysis was performed using clone Alme-1 (Adipogen) mouse anti-RIG-I antibody.

### Limited trypsin digestion.

Confluent 10-cm-diameter dishes of Huh7 or doxycycline-induced 293TdoxRIG-I cells were scraped in binding buffer (25 mM Tris HCl [pH 7.5], 150 mM NaCl, 1.5 mM MgCl_2_) supplemented with 0.5% Triton X-100 without protease inhibitors. The concentration of total protein in clarified lysates was determined by Bradford assay. The reaction volume was 500 µl and included 1 mg to 2 mg total lysate protein, RNA (33 nM to 1 µM), and 2 mM AMP-PNP (Roche) or ATP (New England Biolabs). The reaction mixtures were incubated for 30 min at room temperature to permit RIG-I:RNA complex formation. Next, tosylsulfonyl phenylalanyl chloromethyl ketone (TPCK)-treated trypsin (Sigma-Aldrich) was added to each reaction for a final mass ratio of 1:400, and the reaction mixture was incubated at room temperature. At the indicated time points (0 to 360 min), 25-µl aliquots were removed for boiling in SDS sample buffer. Experiments using multiple RNA concentrations were set up by serially diluting the RNA in binding buffer, with digestions performed for 1.5 h with a 1:200 mass ratio of trypsin. RIG-I fragments were visualized by immunoblotting using clone Alme-1 (Adipogen) mouse anti-RIG-I helicase antibody or monoclonal mouse anti-RIG-I CARDs (KeraFast).

## SUPPLEMENTAL MATERIAL

Figure S1  Transcription of polyU/UC RNA containing modified nucleotides. (A) Sequence of polyU/UC, with the first three guanidine residues added for improved T7 transcription efficiency. (B) Structures of modified nucleotides used in *in vitro* transcription (IVT) reactions with 100% substitution of a modified nucleotide triphosphate for its canonical equivalent. Modifications are indicated in red. “P3” indicates triphosphate. (C) Representative capillary gel electrophoresis of 30 to 80 ng of purified IVT long dsRNA, or polyU/UC RNA containing canonical nucleotides (can) or the indicated modified nucleotides, and software-generated single-stranded RNA (ssRNA) ladder (ld) to determine lengths (25 to 6,000 nt). (D) Representative dot blot for dsRNA, with positive control (dsRNA) and polyU/UC RNA containing canonical nucleotides (can) or the indicated modified nucleotides. Download Figure S1, EPS file, 1.8 MB

Figure S2  Dual-luciferase controls. (A) Huh7 or Huh7.5 cells were transfected with luciferase reporter plasmids as described for Fig. 1A. Cells were then stimulated with polyU/UC RNA containing canonical nucleotides (can) or the indicated modifications, with phosphatase treatment (CIP) or without phosphatase treatment (5′ppp), or with long 5′ppp dsRNA. Results of a representative experiment are shown. The statistical table presents *P* values of results of paired two-tailed *t* tests comparing fold IFN responses determined under the indicated conditions from multiple independent experiments. (B) Huh7 cells were transfected with polyU/UC RNA containing canonical nucleotides (can) or the indicated modifications or were mock transfected. (B and C) At 16 to 24 h post-transfection, cells were lysed and levels of MDA5 and IFIT1 induction were detected by immunoblotting, with GAPDH (glyceraldehyde-3-phosphate dehydrogenase) probed as a gel loading control. IFIT1 induction was not detectable in Huh7.5 cells. (C) Huh7.5 cells were transfected with long dsRNA or with polyU/UC RNA. Download Figure S2, EPS file, 1.7 MB

Figure S3  Quantification of viral infection by flow cytometry. (A and B) Huh7 cells were pretreated with polyU/UC RNA containing canonical nucleotides (can) or the indicated modifications or were mock transfected or untreated. At 16 h-posttransfection, cells were challenged with viral infection. (A) Huh7 cells were analyzed for GFP expression by live-cell flow cytometry at 6 h postinfection with VSV-GFP (MOI of 3). (B) Huh7 cells were analyzed at 36 to 48 h postinfection with dengue virus (MOI of 1). Dengue-infected cells were fixed and permeabilized for immunofluorescent staining to detect DenV NS1 protein. (A and B) Mock-infected conditions are indicated with pink (A) or orange (B) histograms; virus-infected conditions are indicated with green histograms. The number in each panel represents the percentage of positive staining cells under the infected conditions. The data are representative of the results of at least two independent experiments. Download Figure S3, EPS file, 1.6 MB

Figure S4  Stability of modified RNA in cell extract. (A to C) RNA was transcribed with [α-^32^P]CTP radiolabeling and the indicated nucleotide modifications and incubated in cell extracts. (A) Top: total RNA was isolated at the indicated number of minutes (0, 15, 30, or 60 min) postaddition to lysate and analyzed on a Tris-borate-EDTA (TBE)–urea gel with SYBR gold staining, alongside an RNA ladder (ld). Bottom: the stability of the ^32^P-RNA was assayed by autoradiography of the dried gel. (B) The RNA signal was quantified from the digital image and normalized to 0 min to control for differences in radiolabeling efficiency. Error bars indicate standard deviations for the results of three independent experiments. (C) Table of the half-life of RNA in cell lysate, 95% confidence intervals (95% CI), and the goodness of fit (*R^2^*) of the exponential decay curve. Download Figure S4, EPS file, 2.7 MB

Figure S5  Filter binding assay competition control. Recombinant RIG-I (150 pmol) was incubated with 1.5 pmol of polyU/UC ^32^P-RNA and with the indicated competitor RNAs at 1-, 2-, 4-, 8-, and 16-fold mass excess. Protein-RNA complexes were captured on a filter membrane for scintillation counting, normalized to the ^32^P-RNA signal in the absence of competitor. Error bars represent standard deviations of the results of technical duplicates. The graph is representative of the results of three independent experiments. Download Figure S5, EPS file, 0.7 MB

Figure S6  Limited trypsin digestion of endogenously expressed RIG-I. Limited trypsin digestion assays were performed with RNA ligands and with AMP-PNP as described for Fig. 3 but with the use of Huh7 cell lysate containing endogenously expressed RIG-I instead of 293T cell lysate with doxycycline-inducible RIG-I. Download Figure S6, EPS file, 2.2 MB

Text S1  Supplemental materials and methods used in HPLC and dot blot assays for dsRNA, RNA stability in cell lysate, and endogenous ISG induction and in RIG-I:RNA filter binding assays. Download Text S1, DOCX file, 0.02 MB
