# Supplementary material for: RNAs Containing Modified Nucleotides Fail To Trigger RIG-I Conformational Changes for Innate Immune Signaling
Source: mBio. 2016 Sep 20;7(5):e00833-16. doi: 10.1128/mBio.00833-16 (PMC5030355; doi:10.1128/mBio.00833-16)
Supplement: Text S1 — Supplemental materials and methods used in HPLC and dot blot assays for dsRNA, RNA stability in cell lysate, and endogenous ISG induction and in RIG-I:RNA filter binding assays. Download [file mbo004162997s7.docx]

Supplemental Experimental Procedures

*HPLC and Dot Blot for dsRNA*

To detect double-stranded contamination amidst the single stranded IVT product, a dot blot protocol was adapted from (K Kariko, H Muramatsu, J Ludwig, D Weissman, Nucleic Acids Res 39:e142, 2011, doi: 10.1093/nar/gkr695). Briefly, RNA preps were bound to a Nytran Supercharge membrane (GE Healthcare), blocked with 5% Blotto (Santa Cruz) in Tis-buffered saline with 0.5% Tween-20 (TBST), and probed with antibody clone J2 (English Scientific), which binds dsRNA longer than 40bp ([35](#_ENREF_35)). Secondary antibody anti-mouse-HRP (Promega) was visualized with ELC Plus substrate (Thermo) in a G:Box Xl1.4 Chemi/Fluoro Imager (Syngene) and digital images were analyzed with GeneSys software (SynGene).

The dsRNA reactivity in spots of 200ng of IVT RNA was compared to the intensity of positive control dsRNA at 25ng, 5ng, and 1ng. Preps of IVT RNA with >99.5% purity were used in experiments, while dsRNA-containing preps were further purified by high performance liquid chromatography (HPLC). HPLC was performed as described by K Kariko and colleagues (K Kariko, H Muramatsu, J Ludwig, D Weissman, Nucleic Acids Res 39:e142, 2011, doi: 10.1093/nar/gkr695) . Briefly, RNA was injected into an RNASep Cartridge (Transgenomic) in tri-ethyl ammonium acetate (0.1M TEAA). RNA was eluted over a gradient of 0% to 25% acetonitrile, automated with a Waters 600E Multisolvent Delivery System. Fractions were collected by hand, and peaks of strong OD260 absorbance were pooled for further purification. Amicon Ultra-15 Centrifugal Filter units (30kDa membrane) were used for buffer exchange of eluted RNA into water. Aqueous RNA was analyzed again by capillary electrophoresis, by Nanodrop 2000 spectrophotometer, and by anti-dsRNA dot blot. RNA was aliquoted and stored at -80^o^C.

*RNA Stability in Cell Lysate*

293TdoxRIG-I cells were cultured without doxycycline induction and harvested in Buffer Z (50mM Tris HCl pH7.5, 150mM NaCl, 2mM MgCl2, 10% glycerol, 1% Triton X-100) supplemented with EDTA-free protease inhibitor (Roche). The protein concentrations of clarified lysates were determined by Bradford assay. Reactions containing 1.5mg of lysate and 3ug of ^32^P-CTP-radiolabeled RNA were incubated at room temperature, with aliquots removed at the indicated time points (0min, 30min, 60min). Total RNA was extracted with Trizol LS Reagent (Thermo) according to the manufacturer’s instructions and precipitated with isopropanol. Reconstituted total RNA was loaded into a 10% TBE-Urea gel (Novex) at 2ug per lane in formamide-based 2x RNA Loading Dye (New England BioLabs). Gels were stained with SYBR Gold nucleic acid dye (Invitrogen) for imaging on the UV illuminated box of the G:Box Xl1.4 Chemi/Fluoro Imager (Syngene), for analysis with GeneSys software. Gels were dried on filter paper at 70^o^C with vacuum in a Slab-Gel Drier 4050 (Savant). A phospho-screen (Amersham Biosciences) was exposed to the gel in a dark cassette for 1 - 5 days and imaged in a Typhoon FLA 7000 phosphoimager (GE Healthcare), and digital images were analyzed with GeneSys software.

The signal from radiolabeled RNA was normalized per unit of cellular RNA, and then normalized relative to the 0min time point. Data were analyzed in GraphPad Prism 6, using an one-phase exponential best-fit curve: Y=(Y0)*exp(-K*T). The fraction of radiolabeled-RNA (Y) at time 0min was normalized to 100% (Y0), with time (T) measured in minutes, and half life defined as (1/K)*ln(2).
*Endogenous ISG Induction*

Huh7 cells were seeded in 12-well plate at 4 x 10^5 cells/well. The next day, cells were transfected with 1.5ug/well RNA, using LipoJet transfection reagent (SignaGen) according the manufacturer’s instructions. At 24hr, cells were lysed in Buffer Z (50mM Tris HCl pH7.5, 150mM NaCl, 2mM MgCl2, 10% glycerol, 1% Triton X-100) supplemented with EDTA-free protease inhibitor (Roche). Lysates were clarified by centrifugation and boiled in SDS sample buffer prior to loading into a 10% Tris-glycine polyacrylamide gel (Thermo, Precise brand) for SDS-PAGE. The samples were transferred onto nitrocellulose membrane (Thermo), and blocked with 5% Blotto (Santa Cruz) in TBST buffer. The membrane was probed with anti-MDA5 (Neobiolabs), anti-IFIT1 (Origene), and anti-GAPDH (Santa Cruz).

*RIG-I:RNA Filter Binding Assay*

Filter binding assay was designed around the principles established previously (OC Uhlenbeckm, J Carey, PJ Romaniuk, PT Lowary, D Beckett, J Biomol Struct Dyn 1:539-552, 1983, doi: 10.1080/07391102.1983.10507460). Recombinant RIG-I was present at up to 1000 fold molar excess over polyU/UC RNA. RIG-I was serially diluted 1:3 in 18ul Binding buffer (25mM Tris HCl pH7.5, 150mM NaCl, 1.5mM MgCl_2_), then 7ul of an RNA:BSA mixture in Binding Buffer was added, for a 25ul final reaction volume, with 1pmole of RNA (46nM), 600nM BSA and 0-50nM RIG-I. Reactions were incubated at room temperature for 30min to allow the RNA:RIG-I complexes to form. Reactions were then diluted in 500ul of Binding buffer and applied to a .45um nitrocellulose membrane filter (Millipore) under vacuum filtration to trap RIG-I:RNA complexes. Filters were washed with Binding buffer, air dried, and read in 10ml Opti-Fluor scintillation fluid in a Tri-Carb 2800 instrument (Perkin Elmer) in duplicate reads. The signal recorded in reactions with radiolabeled RNA and no RIG-I was considered background and subtracted from all the samples.

The maximum radiolabeled RNA signal observed in a RIG-I containing reaction was normalized to 100%. The logarithm of the RIG-I concentration was plotted against the fraction of RNA bound, combining at least three independent experiments per ligand, for a total of at least 34 data points per ligand. In GraphPad Prism 6 software, we performed non-linear regression using the equation Y = 100/(1+10^((LogKd - X)*Slope)). Y is the normalized fraction of RNA bound, X is the concentration of RIG-I, and the Slope is a unit-less parameter. The RIG-I concentration at which 50% of the RNA is RIG-I-bound was taken as the dissociation constant (Kd). An extra-sum-of-squares F-test was used to test if an RNA*mod* binding curve was best fit by the RNA*can* binding curve (null hypothesis, shared parameters) or best-fit by a second curve (alternative hypothesis, independent Slope and Kd parameters). The P value indicates the probability that, for a data set of the same size, biologically explained by a single curve, the improved sum-of-squares observed in the two-curve model would occur by chance, with p<0.05 set as the significance cut off.
